# {(*E*)-2-[3-(Dimethyl­ammonio)propyl­iminometh­yl]phenolato}diiodidozinc(II)

**DOI:** 10.1107/S1600536808023659

**Published:** 2008-07-31

**Authors:** Xue-Wen Zhu, Xu-Zhao Yang

**Affiliations:** a Key Laboratory of Surface and Interface Science of Henan, School of Materials and Chemical Engineering, Zhengzhou University of Light Industry, Zhengzhou 450002, People’s Republic of China

## Abstract

The title complex, [ZnI_2_(C_12_H_18_N_2_O)], is a mononuclear zinc(II) compound derived from the zwitterionic form of the Schiff base (*E*)-2-[(3-dimethyl­amino­propyl­imino)meth­yl]phenol. The Zn^II^ atom is four-coordinated by the imine N and phenolate O atoms of the Schiff base ligand, and by two iodide ions in a tetra­hedral coordination geometry. In the crystal structure, mol­ecules are linked through inter­molecular N—H⋯O hydrogen bonds, forming chains running along the *b* axis.

## Related literature

For background to the chemistry of Schiff base complexes, see: Ali *et al.* (2008 ▶); Biswas *et al.* (2008 ▶); Chen *et al.* (2008 ▶); Darensbourg & Frantz (2007 ▶); Habibi *et al.* (2007 ▶); Kawamoto *et al.* (2008 ▶); Lipscomb & Sträter (1996 ▶); Tomat *et al.* (2007 ▶); Wu *et al.* (2008 ▶); Yuan *et al.* (2007 ▶). For related structures, see: Qiu (2006*a* ▶,*b* ▶); Wei *et al.* (2007 ▶); Zhu *et al.* (2007 ▶).
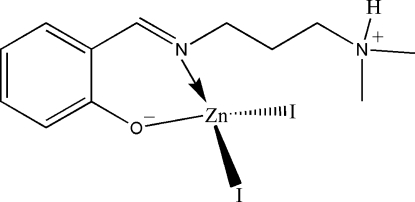


## Experimental

### 

#### Crystal data

[ZnI_2_(C_12_H_18_N_2_O)]
*M* *_r_* = 525.45
Orthorhombic, 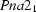

*a* = 13.892 (3) Å
*b* = 16.640 (2) Å
*c* = 7.372 (3) Å
*V* = 1704.1 (8) Å^3^
*Z* = 4
Mo *K*α radiation
μ = 5.06 mm^−1^
*T* = 298 (2) K
0.20 × 0.20 × 0.18 mm

#### Data collection

Bruker APEXII CCD area-detector diffractometer
Absorption correction: multi-scan (*SADABS*; Sheldrick, 2004 ▶) *T*_min_ = 0.431, *T*_max_ = 0.463 (expected range = 0.375–0.402)
12154 measured reflections
3669 independent reflections
3271 reflections with *I* > 2σ(*I*)
*R*_int_ = 0.048

#### Refinement

*R*[*F* ^2^ > 2σ(*F* ^2^)] = 0.043
*wR*(*F* ^2^) = 0.100
*S* = 1.04
3669 reflections
165 parameters
1 restraint
H-atom parameters constrained
Δρ_max_ = 1.82 e Å^−3^
Δρ_min_ = −0.47 e Å^−3^
Absolute structure: Flack (1983 ▶), 1660 Friedel pairs
Flack parameter: 0.00 (4)

### 

Data collection: *APEX2* (Bruker, 2004 ▶); cell refinement: *SAINT* (Bruker, 2004 ▶); data reduction: *SAINT*; program(s) used to solve structure: *SHELXS97* (Sheldrick, 2008 ▶); program(s) used to refine structure: *SHELXL97* (Sheldrick, 2008 ▶); molecular graphics: *SHELXTL* (Sheldrick, 2008 ▶); software used to prepare material for publication: *SHELXTL*.

## Supplementary Material

Crystal structure: contains datablocks global, I. DOI: 10.1107/S1600536808023659/sj2522sup1.cif

Structure factors: contains datablocks I. DOI: 10.1107/S1600536808023659/sj2522Isup2.hkl

Additional supplementary materials:  crystallographic information; 3D view; checkCIF report

## Figures and Tables

**Table:** 

Zn1—O1	1.952 (4)
Zn1—N1	2.010 (6)
Zn1—I2	2.5550 (11)
Zn1—I1	2.5615 (11)

**Table:** 

O1—Zn1—N1	94.3 (2)
O1—Zn1—I2	112.17 (16)
N1—Zn1—I2	113.02 (16)
O1—Zn1—I1	112.90 (16)
N1—Zn1—I1	106.74 (18)
I2—Zn1—I1	115.67 (4)

**Table 2:** Hydrogen-bond geometry (Å, °)

*D*—H⋯*A*	*D*—H	H⋯*A*	*D*⋯*A*	*D*—H⋯*A*
N2—H2*A*⋯O1^i^	0.91	1.91	2.772 (8)	157

Symmetry code: (i) 
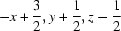
.

**Fig. 1.**
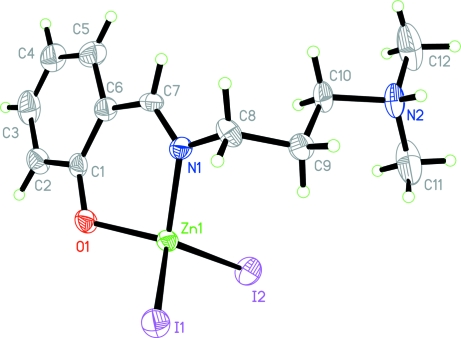
The molecular structure of (I) with ellipsoids drawn at the 30% probability level.

**Fig. 2.**
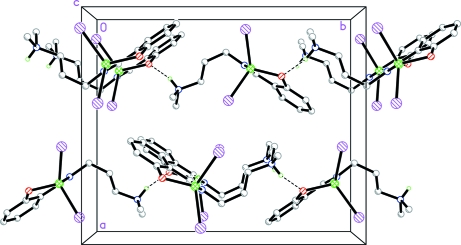
The crystal packing of (I), viewed along the c axis.

## supplementary crystallographic information

### Comment

Schiff bases have widely been used as versatile ligands in coordination chemistry (Biswas *et al.*, 2008; Wu *et al.*, 2008; Kawamoto *et al.*, 2008; Ali *et al.*, 2008; Habibi *et al.*, 2007), and their metal complexes are of great interest in many fields (Chen *et al.*, 2008; Yuan *et al.*, 2007; Tomat *et al.*, 2007; Darensbourg & Frantz, 2007). Zinc(II) is an important element in biological systems and functions as the active site of hydrolytic enzymes, such as carboxypeptidase and carbonic anhydrase  where it is in a hard-donor coordination environment of nitrogen and oxygen ligands (Lipscomb & Sträter, 1996). In this paper, a new zinc(II) complex, (I), Fig. 1, of the Schiff base ligand (E)-2-[(3-dimethylaminopropylimino)methyl]phenol has been synthesized and structurally characterized.

The Zn^II^ atom in (I) is four-coordinated by the imine N and phenolate O atoms of the zwitterionic form of the Schiff base ligand, and by two I^-^ ions, in a tetrahedral coordination geometry. The coordinate bond lengths (Table 1) are typical and comparable to the corresponding values observed in other similar zinc(II) Schiff base complexes (Zhu *et al.*, 2007; Wei *et al.*, 2007; Qiu, 2006*a*,b).

In the crystal structure, molecules are linked through intermolecular N–H···O hydrogen bonds (Table 2), forming chains running along the *b* axis (Fig. 2).

### Experimental

The Schiff base compound was prepared by the condensation of equimolar amounts of salicylaldehyde with *N*,*N*-dimethylpropane-1,3-diamine in a methanol solution. The complex was prepared by the following method. To an anhydrous methanol solution (5 ml) of ZnI_2_ (31.9 mg, 0.1 mmol) was added a methanol solution (10 ml) of the Schiff base compound (20.6 mg, 0.1 mmol) with stirring. The mixture was stirred for 30 min at room temperature and filtered. Upon keeping the filtrate in air for a few days, colorless block-shaped crystals were formed at the bottom of the vessel on slow evaporation of the solvent.

### Refinement

All H atoms were placed in geometrically idealized positions and constrained to ride on their parent atoms, with C–H distances in the range 0.93–0.97 Å, N–H distances of 0.91 Å, and with *U*_iso_(H) = 1.2*U*_eq_(C,*N*) and 1.5*U*_eq_(methyl C).

### Crystal data

**Table:**

[ZnI_2_(C_12_H_18_N_2_O)]	*F*_000_ = 992
*M**_r_* = 525.45	*D*_x_ = 2.048 Mg m^−^^3^
Orthorhombic, *P**n**a*2_1_	Mo *K*α radiation λ = 0.71073 Å
Hall symbol: P 2c -2n	Cell parameters from 4125 reflections
*a* = 13.892 (3) Å	θ = 2.4–25.0º
*b* = 16.640 (2) Å	µ = 5.06 mm^−^^1^
*c* = 7.372 (3) Å	*T* = 298 (2) K
*V* = 1704.1 (8) Å^3^	Block, colorless
*Z* = 4	0.20 × 0.20 × 0.18 mm

### Data collection

**Table:**

Bruker APEXII CCD area-detector diffractometer	3669 independent reflections
Radiation source: fine-focus sealed tube	3271 reflections with *I* > 2σ(*I*)
Monochromator: graphite	*R*_int_ = 0.048
*T* = 298(2) K	θ_max_ = 27.0º
ω scans	θ_min_ = 1.9º
Absorption correction: multi-scan(SADABS; Sheldrick, 2004)	*h* = −17→17
*T*_min_ = 0.431, *T*_max_ = 0.463	*k* = −20→21
12154 measured reflections	*l* = −9→9

### Refinement

**Table:**

Refinement on *F*^2^	Hydrogen site location: inferred from neighbouring sites
Least-squares matrix: full	H-atom parameters constrained
*R*[*F*^2^ > 2σ(*F*^2^)] = 0.043	*w* = 1/[σ^2^(*F*_o_^2^) + (0.0426*P*)^2^ + 0.9395*P*] where *P* = (*F*_o_^2^ + 2*F*_c_^2^)/3
*wR*(*F*^2^) = 0.100	(Δ/σ)_max_ = 0.001
*S* = 1.04	Δρ_max_ = 1.82 e Å^−^^3^
3669 reflections	Δρ_min_ = −0.46 e Å^−^^3^
165 parameters	Extinction correction: none
1 restraint	Absolute structure: Flack (1983), 1660 Friedel pairs
Primary atom site location: structure-invariant direct methods	Flack parameter: 0.00 (4)
Secondary atom site location: difference Fourier map	

### Special details

**Table:**

Geometry. All e.s.d.'s (except the e.s.d. in the dihedral angle between two l.s. planes) are estimated using the full covariance matrix. The cell e.s.d.'s are taken into account individually in the estimation of e.s.d.'s in distances, angles and torsion angles; correlations between e.s.d.'s in cell parameters are only used when they are defined by crystal symmetry. An approximate (isotropic) treatment of cell e.s.d.'s is used for estimating e.s.d.'s involving l.s. planes.
Refinement. Refinement of *F*^2^ against ALL reflections. The weighted *R*-factor *wR* and goodness of fit *S* are based on *F*^2^, conventional *R*-factors *R* are based on *F*, with *F* set to zero for negative *F*^2^. The threshold expression of *F*^2^ > σ(*F*^2^) is used only for calculating *R*-factors(gt) *etc*. and is not relevant to the choice of reflections for refinement. *R*-factors based on *F*^2^ are statistically about twice as large as those based on *F*, and *R*- factors based on ALL data will be even larger.

### Fractional atomic coordinates and isotropic or equivalent isotropic displacement parameters (Å^2^)

**Table:**

	*x*	*y*	*z*	*U*_iso_*/*U*_eq_	
Zn1	0.75194 (6)	0.90239 (4)	0.72869 (14)	0.03631 (19)	
I1	0.58993 (3)	0.92236 (3)	0.88834 (9)	0.04992 (15)	
I2	0.89258 (4)	0.98333 (3)	0.85716 (9)	0.05741 (18)	
N1	0.7294 (4)	0.9228 (3)	0.4634 (8)	0.0379 (13)	
N2	0.8361 (5)	1.1716 (3)	0.3321 (11)	0.0544 (19)	
H2A	0.7857	1.2052	0.3106	0.065*	
O1	0.7847 (4)	0.7893 (3)	0.6946 (7)	0.0429 (12)	
C1	0.8309 (5)	0.7664 (4)	0.5451 (11)	0.0385 (16)	
C2	0.8869 (6)	0.6960 (5)	0.5496 (14)	0.053 (2)	
H2	0.8923	0.6672	0.6573	0.063*	
C3	0.9338 (6)	0.6691 (5)	0.396 (2)	0.070 (3)	
H3	0.9722	0.6234	0.4030	0.084*	
C4	0.9256 (7)	0.7066 (5)	0.2392 (16)	0.063 (3)	
H4	0.9575	0.6865	0.1380	0.075*	
C5	0.8701 (7)	0.7758 (5)	0.2225 (15)	0.063 (2)	
H5	0.8653	0.8019	0.1113	0.075*	
C6	0.8209 (5)	0.8061 (4)	0.3760 (13)	0.0422 (15)	
C7	0.7648 (5)	0.8782 (4)	0.3445 (10)	0.0389 (16)	
H7	0.7542	0.8926	0.2243	0.047*	
C8	0.6691 (5)	0.9919 (4)	0.4056 (12)	0.0456 (19)	
H8A	0.6074	0.9889	0.4667	0.055*	
H8B	0.6574	0.9880	0.2761	0.055*	
C9	0.7143 (6)	1.0712 (4)	0.4458 (11)	0.0459 (19)	
H9A	0.7358	1.0721	0.5710	0.055*	
H9B	0.6668	1.1134	0.4305	0.055*	
C10	0.7992 (6)	1.0871 (4)	0.3217 (13)	0.049 (2)	
H10A	0.8510	1.0505	0.3531	0.059*	
H10B	0.7802	1.0758	0.1977	0.059*	
C11	0.8730 (10)	1.1894 (6)	0.5117 (19)	0.100 (5)	
H11A	0.9164	1.1476	0.5488	0.150*	
H11B	0.8205	1.1926	0.5960	0.150*	
H11C	0.9066	1.2398	0.5092	0.150*	
C12	0.9089 (8)	1.1848 (6)	0.186 (2)	0.101 (5)	
H12A	0.9269	1.2405	0.1834	0.151*	
H12B	0.8819	1.1699	0.0711	0.151*	
H12C	0.9648	1.1525	0.2099	0.151*	

### Atomic displacement parameters (Å^2^)

**Table:**

	*U*^11^	*U*^22^	*U*^33^	*U*^12^	*U*^13^	*U*^23^
Zn1	0.0426 (5)	0.0328 (3)	0.0335 (4)	0.0041 (3)	0.0026 (4)	0.0028 (3)
I1	0.0415 (3)	0.0491 (2)	0.0591 (3)	0.00396 (18)	0.0126 (3)	−0.0002 (3)
I2	0.0486 (3)	0.0603 (3)	0.0633 (4)	−0.0039 (2)	−0.0084 (3)	−0.0062 (3)
N1	0.037 (3)	0.042 (3)	0.035 (3)	−0.012 (2)	−0.001 (3)	−0.004 (3)
N2	0.048 (4)	0.029 (3)	0.086 (6)	0.005 (3)	−0.002 (4)	0.007 (3)
O1	0.052 (3)	0.032 (2)	0.045 (3)	0.008 (2)	0.007 (3)	0.006 (2)
C1	0.034 (4)	0.033 (3)	0.048 (5)	−0.008 (3)	0.005 (3)	−0.002 (3)
C2	0.055 (5)	0.040 (4)	0.063 (6)	0.008 (4)	0.016 (4)	0.005 (4)
C3	0.059 (5)	0.040 (4)	0.111 (9)	0.008 (4)	0.019 (7)	−0.003 (6)
C4	0.063 (6)	0.052 (5)	0.073 (7)	0.010 (4)	0.028 (5)	−0.016 (5)
C5	0.070 (6)	0.063 (5)	0.055 (6)	0.001 (4)	0.014 (5)	−0.012 (5)
C6	0.040 (4)	0.040 (3)	0.046 (4)	0.003 (3)	0.008 (4)	−0.004 (4)
C7	0.049 (4)	0.044 (3)	0.024 (4)	0.001 (3)	0.003 (3)	0.001 (3)
C8	0.041 (4)	0.045 (3)	0.051 (5)	0.001 (3)	−0.004 (4)	0.017 (4)
C9	0.051 (5)	0.039 (4)	0.047 (5)	0.016 (3)	−0.006 (4)	0.005 (3)
C10	0.051 (5)	0.027 (3)	0.070 (6)	0.005 (3)	0.005 (4)	0.006 (3)
C11	0.111 (10)	0.054 (6)	0.135 (11)	−0.023 (6)	−0.078 (9)	0.026 (6)
C12	0.074 (7)	0.052 (5)	0.177 (15)	0.007 (5)	0.051 (8)	0.028 (8)

### Geometric parameters (Å, °)

**Table:**

Zn1—O1	1.952 (4)	C5—C6	1.415 (12)
Zn1—N1	2.010 (6)	C5—H5	0.9300
Zn1—I2	2.5550 (11)	C6—C7	1.449 (9)
Zn1—I1	2.5615 (11)	C7—H7	0.9300
N1—C7	1.250 (9)	C8—C9	1.491 (10)
N1—C8	1.485 (9)	C8—H8A	0.9700
N2—C11	1.451 (14)	C8—H8B	0.9700
N2—C12	1.493 (15)	C9—C10	1.516 (12)
N2—C10	1.498 (8)	C9—H9A	0.9700
N2—H2A	0.9100	C9—H9B	0.9700
O1—C1	1.331 (9)	C10—H10A	0.9700
C1—C2	1.406 (10)	C10—H10B	0.9700
C1—C6	1.417 (12)	C11—H11A	0.9600
C2—C3	1.384 (15)	C11—H11B	0.9600
C2—H2	0.9300	C11—H11C	0.9600
C3—C4	1.317 (17)	C12—H12A	0.9600
C3—H3	0.9300	C12—H12B	0.9600
C4—C5	1.392 (12)	C12—H12C	0.9600
C4—H4	0.9300		
			
O1—Zn1—N1	94.3 (2)	N1—C7—C6	126.2 (7)
O1—Zn1—I2	112.17 (16)	N1—C7—H7	116.9
N1—Zn1—I2	113.02 (16)	C6—C7—H7	116.9
O1—Zn1—I1	112.90 (16)	N1—C8—C9	113.0 (6)
N1—Zn1—I1	106.74 (18)	N1—C8—H8A	109.0
I2—Zn1—I1	115.67 (4)	C9—C8—H8A	109.0
C7—N1—C8	118.8 (7)	N1—C8—H8B	109.0
C7—N1—Zn1	121.4 (5)	C9—C8—H8B	109.0
C8—N1—Zn1	119.9 (5)	H8A—C8—H8B	107.8
C11—N2—C12	112.8 (9)	C8—C9—C10	111.2 (6)
C11—N2—C10	111.1 (7)	C8—C9—H9A	109.4
C12—N2—C10	109.5 (8)	C10—C9—H9A	109.4
C11—N2—H2A	107.8	C8—C9—H9B	109.4
C12—N2—H2A	107.8	C10—C9—H9B	109.4
C10—N2—H2A	107.8	H9A—C9—H9B	108.0
C1—O1—Zn1	119.6 (4)	N2—C10—C9	113.5 (6)
O1—C1—C2	119.0 (7)	N2—C10—H10A	108.9
O1—C1—C6	123.2 (6)	C9—C10—H10A	108.9
C2—C1—C6	117.6 (7)	N2—C10—H10B	108.9
C3—C2—C1	120.7 (9)	C9—C10—H10B	108.9
C3—C2—H2	119.6	H10A—C10—H10B	107.7
C1—C2—H2	119.6	N2—C11—H11A	109.5
C4—C3—C2	121.6 (8)	N2—C11—H11B	109.5
C4—C3—H3	119.2	H11A—C11—H11B	109.5
C2—C3—H3	119.2	N2—C11—H11C	109.5
C3—C4—C5	121.2 (9)	H11A—C11—H11C	109.5
C3—C4—H4	119.4	H11B—C11—H11C	109.5
C5—C4—H4	119.4	N2—C12—H12A	109.5
C4—C5—C6	119.5 (10)	N2—C12—H12B	109.5
C4—C5—H5	120.3	H12A—C12—H12B	109.5
C6—C5—H5	120.3	N2—C12—H12C	109.5
C5—C6—C1	119.3 (7)	H12A—C12—H12C	109.5
C5—C6—C7	115.2 (8)	H12B—C12—H12C	109.5
C1—C6—C7	125.4 (7)		

### Hydrogen-bond geometry (Å, °)

**Table:**

*D*—H···*A*	*D*—H	H···*A*	*D*···*A*	*D*—H···*A*
N2—H2A···O1^i^	0.91	1.91	2.772 (8)	157

Symmetry codes: (i) −*x*+3/2, *y*+1/2, *z*−1/2.
